# Concurrent changes in dental anxiety and smoking in parents of the FinnBrain Birth Cohort Study

**DOI:** 10.1111/eos.12912

**Published:** 2023-01-04

**Authors:** Anu Kallio, Auli Suominen, Mimmi Tolvanen, Kari Rantavuori, Heidi Jussila, Linnea Karlsson, Hasse Karlsson, Satu Lahti

**Affiliations:** ^1^ Department of Community Dentistry University of Turku Turku Finland; ^2^ City of Turku Welfare Services Division Oral and Dental Health Care Turku Finland; ^3^ Center for Life Course Health Research University of Oulu Oulu Finland; ^4^ Department of Oral Development and Orthodontics University of Turku Turku Finland; ^5^ Cleft Palate and Craniofacial Center Department of Plastic Surgery Helsinki University Hospital and Helsinki University Helsinki Finland; ^6^ FinnBrain Birth Cohort Study Department of Clinical Medicine Brain and Mind Center University of Turku Turku Finland; ^7^ Department of Psychiatry University of Turku and Turku University Hospital Turku Finland; ^8^ Centre for Population Health Research University of Turku Turku Finland

**Keywords:** cohort study, longitudinal study, pregnancy

## Abstract

We evaluated associations between changes in dental anxiety and tobacco use, adjusted for general anxiety and depressive symptoms. The FinnBrain Birth Cohort Study data, collected at gestational weeks 14 and 34 and at 3 months postpartum, were used. Questionnaires included the Modified Dental Anxiety Scale (MDAS), the Edinburgh Postnatal Depression Scale (EPDS), and the anxiety subscale of the Symptom Checklist‐90 (SCL). Smoking was categorized as “stable non‐smoking”, “started smoking”, “quit smoking”, and “stable smoking”. Changes in smoking and dental anxiety were evaluated “during pregnancy” (i.e., from gestational week 14 to gestational week 34) in 2442 women and 1346 men and “after pregnancy” (i.e., from gestational week 34 to 3 months postpartum) in 2008 women and 1095 men. Changes were evaluated in three smoking categories (stable non‐smoking, fluctuating, and stable smoking), using data from all three time‐points (1979 women and 1049 men). Modeling used repeated measures analysis of covariance. Stable smoking mothers had statistically significantly higher levels of dental anxiety (mean MDAS 12.3–12.6) than non‐smoking mothers (mean MDAS 10.1–10.7) or mothers who smoked at some point during pregnancy (mean MDAS 10.8–11.5). A similar tendency was observed in fathers. However, no systematic change in dental anxiety by changes in smoking habits was observed. Those smoking during pregnancy and with high dental anxiety may need special support for smoking cessation.

## INTRODUCTION

Dental fear and anxiety (henceforth referred to as dental anxiety) are prevalent problems in Finland and worldwide, affecting up to 50% of adults and children [1, 2].Dental anxiety is associated with delay or avoidance of dental treatment and, consequently, with poor oral health and quality of life [1, 3‐6]. The underlying causes of dental anxiety are multifactorial, usually categorized as exogenous (direct or vicarious experiences) or endogenous (e.g., temperament or vulnerability to anxiety disorders) [7, 8]. Dental anxiety also shows differences by age, gender, education, and socio‐economic status [1, 9‐16].

When considering oral health‐related behaviors aside from oral health service visiting patterns, the use of tobacco products has been shown to be associated with high dental anxiety [14, 15, 17‐19]; this association is more consistent than that between dental anxiety and oral self‐care [20, 21].Adult and adolescent smokers have higher rates of dental anxiety than non‐smokers [3, 15, 16, 22, 23]. However, the underlying causes behind this connection between dental anxiety and smoking are currently not well known; common vulnerability has been suggested as one explanation, along with genetic factors [24, 25, 26].

Constitutional vulnerability has been suggested as one explanation [9] for the associations found between dental anxiety and psychological disorders, such as phobias and other anxiety disorders, mood disorders, and substance use [27, 28, 29, 30, 31, 32, 33, 34, 35, 36, 37, 38, 39, 40, 41, 42, 43, 44]. Moreover, tobacco use and nicotine dependence have been shown to be associated with depression and general anxiety [45, 46, 47, 48, 49, 50, 51, 52, 53, 54, 55].

To our knowledge, no longitudinal studies have examined concurrent changes in smoking and dental anxiety, possibly because it is difficult to find a sufficiently large population where smoking cessation within a given period is taking place. However, many parents (especially mothers) quit smoking during pregnancy. A proportion remain non‐smokers, but some return to smoking after childbirth [56]. The FinnBrain Cohort Study (collecting data from parents since early pregnancy) provided a possibility to examine the association between changes in dental anxiety and tobacco use. Thus, our aim was to study whether changes in smoking and dental anxiety occur concurrently.

## MATERIAL AND METHODS

### Settings and design

This is a secondary analysis of the longitudinal data from the FinnBrain Birth Cohort Study (www.finnbrain.fi), which prospectively studies the effects of environment and genes on children´s brain development and health [57]. Participants were recruited in 2011–2015 after the ultrasonography appointments that are offered free of charge to every pregnant mother in Finland by municipal maternity clinics during the first trimester of the pregnancy (gestational week 12) in the South‐Western Hospital District and the Åland Islands in Finland. The coverage of these appointments attended by parents is close to 100% in the population at gestational week 12 [57, 58]. The Ethics Committee of the Hospital District of Southwest Finland has approved the study protocol (14.6.2011 ETMK: 57/180/2011 § 168). Written informed consent was obtained from all participants.

### Sample selection

Out of 8895 newly pregnant women visiting the recruitment sites, 5790 could be reached and informed. Partners of the women were recruited either at the site or by the women at home. Of those informed about the study, a total of 3808 (66%) women and 2623 men answered the questions concerning both dental anxiety and smoking at gestational week 14, and started the study. First, we monitored changes in smoking and dental anxiety at two periods: “during pregnancy” (from gestational week 14 to gestational week 34) and “after pregnancy” (from gestational week 34 to 3 months postpartum). Complete data on dental anxiety and smoking were available “during pregnancy” for 2442 (42%) women and 1346 men, and for 2008 (35%) women and 1095 men “after pregnancy”.

Second, we investigated changes in dental anxiety scores in three smoking groups (stable non‐smoking, fluctuating, and stable smoking) and at three time‐points for the 1979 women and 1049 men who had complete data at all three timepoints (gestational weeks 14 and 34 and at 3 months postpartum). The three smoking categories were considered as ordinal.

### Measuring tools

Dental anxiety was evaluated with the Finnish translation of the Modified Dental Anxiety Scale (MDAS), a valid and widely used five‐item instrument for self‐rating dental anxiety [59, 60, 61]. Each item has five response alternatives, with scores ranging from 1 (not anxious) to 5 (extremely anxious); summated scores range from 5 to 25. The MDAS was used at gestational weeks 14 and 34, and at 3 months after pregnancy. In addition, we calculated an MDAS change score from each previous time point. If there were ≤30% missing items for the MDAS, they were imputed with the mean value of the answered items. These imputations were done for four mothers and two fathers “during pregnancy” and for three and one “after pregnancy”, respectively.

Smoking was evaluated with three questions at the following timepoints: “Have you smoked during this pregnancy?” (gestational week 14); “Have you smoked during the past 5 months?” (gestational week 34); and “Have you smoked after childbirth?” (at 3 months postpartum). Additionally, information about maternal smoking at gestational weeks 14 and 34 was used from the Finnish Medical Birth Register (FMBR), which provides information standardized data on pregnancy, including the prenatal and neonatal periods, for research on all births in Finland.The register information about smoking has four categories: ”no smoking“, “quit smoking during the first trimester”, “smoking throughout the whole pregnancy”, and “information not available”. We created a binary variable called Smoking (0 = yes, 1 = no), which was affirmative when the mother or father had self‐reported or the mother had a register record of smoking at gestational weeks 14, 34 and at 3 months after pregnancy. Second, we formed a 4‐category ordinal variable (0 = stable non‐smoking, 1 = started smoking, 2 = quit smoking, 3 = stable smoking) representing the change in smoking habits. The change was categorized at two separate intervals, between gestational weeks 14 and 34, and between gestational week 34 and 3 months after pregnancy. For the repeated measurements analysis, a 3‐class variable was created by combining categories 1 and 2 as “fluctuating smoking”.

Depressive symptoms were assessed using the Edinburgh Postnatal Depression Scale (EPDS). The EPDS is a widely used and studied questionnaire that is valid and reliable for screening for both pre‐ and postnatal depression in women and men [62, 63, 64, 65, 66]. The scale consists of 10 questions scored on a 4‐point Likert scale (from 0 to 3). For EPDS, a sum score was used. The higher the score, the more depressive symptoms the respondent had. General anxiety symptoms were measured using the anxiety subscale of the Symptom Checklist −90 anxiety subscale (SCL) [67]. The Finnish version of the SCL has shown to be valid and reliable [68]. The scale consists of 10 items scored on a 5‐point Likert scale (from 0 to 4) and the range of total sum scores is 0–40. For the SCL, a sum score was also used, and higher values indicated more anxiety symptoms [67, 68, 69]. EDPS and SCL were assessed at gestational weeks 14, 34, and at 3 months after pregnancy. If there were ≤30% missing items for the SCL‐90 or EPDS, they were imputed with the mean value of the answered items. In Finland, education is divided into different levels, as follows: compulsory (1−9  years), vocational or secondary general/academic schooling (11−12  years), and polytechnics and university level (over 12 years). In this study, education level was divided into three levels: low (≤12  years), middle (polytechnics), and high (university degree). Education was evaluated at gestational week 14.

### Statistical analysis

The dependent variables were the MDAS score and the MDAS change scores. The independent variable was change in smoking habits. Analyses were stratified by gender. The distributions of MDAS were asymmetrical and nonparametric tests were used, even though the distributions of the changes in MDAS were symmetrical. The statistical significance of the changes in MDAS scores between timepoints gestational weeks 14 and 34, and between timepoints gestational week 34 and 3 months postpartum, was evaluated with Wilcoxon signed‐rank tests. The differences in MDAS scores and change scores according to smoking classes were evaluated with Mann‐Whitney U‐test (smokers vs. non‐smokers) and Kruskal‐Wallis test (4‐class change in smoking habits).

When studying only those who had complete data for all three timepoints, the differences in MDAS scores between the three smoking categories at each timepoint were tested with the Jonckheere‐Terpstra test, and the changes in MDAS scores during the study were assessed with the Friedman test separately for each smoking category.General linear models (GLM) for repeated measures were conducted separately for mothers and fathers. Dependent variables were MDAS at three different timepoints; the between‐participant factors were change in smoking habits and education, and the covariates were depression and anxiety at baseline. The statistical significance level was set at *P* < 0.05. Statistical analyses used IBM SPSS version 27.

## RESULTS

Table 1 presents the sample characteristics: proportion of smokers, mean sum scores of dental anxiety (MDAS), depressive symptoms (EPDS), and general anxiety symptoms (SCL) at time intervals “during pregnancy” and “after pregnancy”. Among mothers, smoking decreased during pregnancy, but increased postpartum. Among fathers, smoking increased during pregnancy, but decreased after delivery.

**TABLE 1:** Mean values (SD) of Dental anxiety (MDAS) scores, depressive symptoms (EPDS) scores, general anxiety symptoms (SCL) scores, and the frequency of smoking and education categories at different time points and according to follow‐up time, by parent

			Mothers	Fathers
During pregnancy		Gestational week	*n* = 2442	*n* = 1346
	MDAS Mean (SD)	Week 14	10.5 (4.6)	9.0 (3.9)
		Week 34	10.3 (4.5)	9.0 (4.0)
	SCL Mean (SD)	Week 14	3.3 (3.9)	2.4 (3.3)
		Week 34	3.2 (3.9)	1.9 (3.2)
	EPDS Mean (SD)	Week 14	5.1 (4.0)	3.6 (3.3)
		Week 34	4.9 (4.0)	3.1(3.3)
	Smoking % (*n*)	Week 14	11.9 (288)	29.5 (397)
		Week 34	4.8 (117)	31.1 (418)
	Education % (*n*)			
	Low	Week 14	35.3 (861)	45.3 (608)
	Middle	Week 14	29.6 (721)	27.8 (373)
	High	Week 14	35.1 (855)	27.0 (362)

		Gestational week/Postpartum	Mothers	Fathers
After pregnancy			*n* = 2008	*n* = 1095
	MDAS Mean (SD)	Week 34	10.3 (4.4)	9.0 (3.9)
		3 months	10.8 (4.7)	9.3 (4.1)
	SCL Mean (SD)	Week 34	3.1 (3.8)	1.9 (3.1)
		3 months	2.6 (3.5)	2.4 (3.6)
	EPDS Mean (SD)	Week 34	4.7 (4.0)	3.0 (3.9)
		3 months	4.2 (3.8)	3.2 (3.4)
	Smoking % (*n*)	Week 34	4.7 (93)	30.5 (334)
		3 months	9.3 (186)	25.2 (276)

Abbreviations: EPDS, Edinburgh Postnatal Depression Scale; MDAS, Modified Dental Anxiety Scale; SCL, Symptom Checklist −90 (anxiety subscale).

The mean age of mothers was 30.5 years (SD 4.5) and, for fathers, it was 32.2 (SD 5.3) at timepoint gestational week 14.

Dental anxiety and its changes according to changes in smoking during and after pregnancy are presented in Table 2 for both mothers and fathers. Among mothers, stable smokers had higher dental anxiety scores than other smoking groups in the period “during pregnancy”. However, the difference was statistically significant only at gestational week 14. Dental anxiety decreased in all smoking groups during the follow‐up, but the change in dental anxiety was statistically significant only among stable non‐smokers. In the period “after pregnancy”, those who quit smoking had higher dental anxiety scores than other smoking groups, the difference being statistically significant compared to those who started smoking. Dental anxiety increased in the “stable smoking” and “started smoking” groups.

**TABLE 2:** Dental anxiety (MDAS) mean scores and their changes according to changes in smoking among mothers and fathers during pregnancy (at gestational weeks 14 and 34) and 3 months postpartum

**During pregnancy**	Gestational week	Mean scores/ *P*‐values	Stable non‐smoking	Stable smoking	Quitted smoking	Started smoking	P(b)	P(c)
Mothers			*n* = 2143	*n* = 107	*n* = 182	*n* = 10		
	14	MDAS	10.4	12.5	11.0	11.4	0.043	0.384
	34	MDAS	10.2	12.2	10.7	10.6	0.123	0.735
		P(a)	0.002	0.157	0.195	0.634		
		MDAS change	−0.2	−0.3	−0.3	−0.8	0.727	0.743
Fathers			*n* = 881	*n* = 350	*n* = 47	*n* = 68		
	14	MDAS	8.8	9.5	8.3	9.9	0.050	0.064
	34	MDAS	8.9	9.2	8.5	9.9	0.332	0.138
		P(a)	0.028	0.019	0.496	0.991		
		MDAS change	0.1	−0.3	0.2	0.0	0.044	0.991

**After pregnancy**	**Gestational week/month postpartum**							
Mothers			*n* = 1806	*n* = 77	*n* = 16	*n* = 109		
	34	MDAS	10.2	11.8	14.0	10.4	0.065	0.040
	3 months	MDAS	10.7	12.4	13.6	10.9	0.141	0.211
		P(a)	<0.001	0.058	0.527	0.029		
		MDAS change	0.6	0.6	−0.4	0.4	0.334	0.056
Fathers			*n* = 736	*n* = 252	*n* = 86	*n* = 25		
	34	MDAS	8.9	9.2	9.7	6.8	0.005	0.001
	3 months	MDAS	9.2	9.6	9.6	6.7	0.004	0.001
		P(a)	<0.001	0.007	0.821	0.680		
		MDAS change	0.3	0.4	−0.1	−0.1	0.387	0.636

p(a): *P*‐values for testing for differences in MDAS mean scores between timepoints, Wilcoxon signed rank test.

p(b): *P*‐values for testing for differences in MDAS mean scores and MDAS mean change between four smoking categories, Kruskal‐Wallis test.

p(c): *P*‐values for testing for differences in MDAS mean scores and MDAS mean change scores between two smoking categories “quit smoking” and “started smoking”, Mann‐Whitney U‐test.

Abbreviation: MDAS, Modified Dental Anxiety Scale.

Among fathers, those who started smoking in the period “during pregnancy” had higher dental anxiety scores than other smoking groups at gestational week 14. Dental anxiety decreased among the “stable non‐smoking” group. The mean changes in MDAS scores differed by smoking category. In the period “after pregnancy”, those who quit smoking had higher MDAS scores than other smoking groups at both gestational week 34 and at 3 months postpartum. Dental anxiety increased in both the “stable non‐smoking” and “stable smoking” groups.

Table 3 presents mean MDAS scores at all three timepoints (gestational weeks 14, 34 and at 3 months postpartum) by the three smoking categories. Among mothers, stable smokers had higher dental anxiety scores than stable non‐smokers or those with fluctuating smoking, though the difference at gestational week 34 was only approaching statistical significance. Dental anxiety fluctuated within other smoking categories, but in the “stable smoking” category, the change was not statistically significant. Among fathers, no clear difference in dental anxiety between smoking groups was observed, and the only statistically significant change in dental anxiety between timepoints was seen in the “stable non‐smoking” group.

**TABLE 3:** Dental anxiety (MDAS) mean scores and their changes according to changes in smoking among mothers and fathers at gestational weeks 14 and 34 and 3 months postpartum

	Gestational week/month postpartum	Stable non‐smoking	Fluctuating smoking	Stable smoking	P(b)
**Mothers**					
		*n* = 1743	*n* = 152	*n* = 84	
MDAS (mean)	14	10.3	11.1	12.6	0.011
	34	10.1	10.8	12.3	0.051
	3 months	10.7	11.5	12.6	0.009
P(a)		< 0.001	< 0.001	0.57	
**Fathers**					
		*n* = 694	*n* = 86	*n* = 269	
MDAS (mean)	14	8.7	9.5	9.4	0.071
	34	8.8	9.3	9.1	0.427
	3 months	9.1	9.4	9.5	0.926
P(a)		<0.001	0.692	0.084	

P(a): *P*‐values for testing for similar MDAS mean changes between timepoints, Friedman test.

P(b): *P*‐values for testing for similar MDAS mean change between three smoking categories, Jonckheere‐Terpstra test.

Abbreviation: MDAS, Modified Dental Anxiety Scale.

**FIGURE 1:**
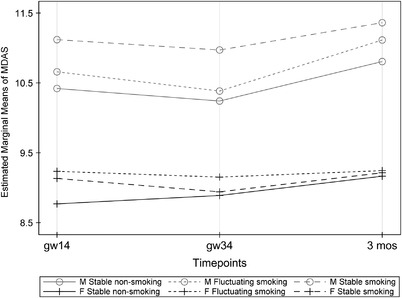
Dental anxiety (MDAS) scores and their changes according to changes in smoking habits among mothers (M) and fathers (F) assessed at gestational weeks 14, 34 and at 3 months postpartum

According to the multivariable model (Table 4), dental anxiety changed during the study among both mothers and fathers. Among fathers, change was not related to change in smoking, or to education, depression, or general anxiety at the baseline; however, among mothers, change in dental anxiety was related to general anxiety at the baseline. The level of dental anxiety was positively associated with depression and general anxiety among both mothers and fathers. Change in smoking was not associated with dental anxiety during the follow‐up (Figure 1).

**TABLE 4:** Regression (beta) coefficients estimated using GLM for repeated measures for the effect of smoking, education, EDPS and SCL scores on MDAS scores at the three timepoints, gestational weeks 14, 34 and 3 months postpartum

Timepoint		Gestational week 14	Gestational week 34	3 months postpartum	P(a)	P(b)
**Mothers**						
Dependent: MDAS at 3 time points					<0.001	
Smoking	stable nonsmoking	−0.7	−0.7	−0.6	0.889	0.346
	fluctuating smoking	−0.5	−0.6	−0.3		
	stable smoking (ref.)	–	–	–		
Education	low	1.4	1.3	1.3	0.969	<0.001
	middle	0.4	0.4	0.4		
	high (ref.)	–	–	–		
EPDS	0.2	0.2	0.2	0.094	<0.001	
SCL	0.1	0.1	0.0	0.002	0.033	
**Fathers**						
Dependent: MDAS at 3 time points					0.019	
Smoking	stable nonsmoking	−0.4	−0.1	−0.1	0.219	0.723
	fluctuating smoking	0.1	0.2	0.0		
	stable smoking (ref.)	–	–	–		
Education	low	0.6	0.4	0.6	0.802	0.128
	middle	0.2	0.2	0.2		
	high (ref.)	–	–	–		
EPDS	0.2	0.2	0.2	0.422	<0.001	
SCL	0.1	0.1	0.1	0.861	0.054	

P(a): *P*‐values for testing the significance of the effect on the change in MDAS score (Covariate*Change in MDAS).

P(b): *P*‐values for testing the significance of the independent effect on MDAS score.

Abbreviations: EPDS, Edinburgh Postnatal Depression Scale; MDAS, Modified Dental Anxiety Scale; SCL, Symptom Checklist −90 (anxiety subscale);

## DISCUSSION

The main finding of this study was that mothers who continue smoking throughout pregnancy have substantially higher levels of dental anxiety than non‐smoking mothers. This difference was also observed among fathers, but not as strongly as in mothers. Moreover, mothers and fathers who smoked at some point in the pregnancy had higher dental anxiety than non‐smoking mothers and fathers. However, the concurrent changes in dental anxiety and smoking were not systematic.

Our findings that adult smokers are more dentally anxious than non‐smokers are in agreement with other studies suggesting an association between use of tobacco products and dental anxiety in the adult population [3, 14, 15, 17‐19]. This association has not been previously studied longitudinally, but our novel finding that those smoking at some point of pregnancy have higher scores of dental anxiety is in accordance with previous cross‐sectional findings.

One of the strengths of this study was its use of a large representative sample; the FinnBrain Birth Cohort sample has been shown to represent well the general population in the geographic area [57]. The data set includes both men and women, and all socio‐economic classes are represented. In addition to a well‐characterized cohort, systematic collection of data using validated questionnaires is another strength. However, the study also has limitations. Smoking was for the first time evaluated at the timepoint of gestational week 14. Thus, information on participants who had quit smoking already in the early weeks of pregnancy has been missed. Also, all measures are self‐reported; for smoking, this can be a source of social desirability bias. Smoking was evaluated with a simple “yes” or “no” question, and this fails to distinguish between an occasional cigarette and regular smoking. Moreover, major life events (such as pregnancy and childbirth) can affect the course of dental anxiety, and so the findings cannot be generalized to non‐pregnant or other populations. The MDAS can capture the three conceptual aspects of dental fear, dental anxiety, and dental phobia [70, 71]. As we use only the term “dental anxiety”, this should be considered when interpreting the findings.

In conclusion, we found no systematic evidence that dental anxiety changed concurrently with changes in smoking in this pregnancy cohort population. In general, those smoking at some point of pregnancy, especially mothers, had higher levels of dental anxiety than non‐smokers. Dental practitioners should pay attention to this when treating child expectant mothers (and fathers) who use tobacco. These findings suggest that dental anxiety and smoking possibly share common background vulnerability factors, but this connection needs to be further studied.

## AUTHOR CONTRIBUTION

**Conceptualization**: Anu Kallio, Satu Lahti; **Methodology**: Anu Kallio, Auli Suominen, Mimmi Tolvanen, Satu Lahti; **Formal analysis**: Auli Suominen, Mimmi Tolvanen; **Investigation**: Linnea Karlsson, Hasse Karlsson; **Writing – original draft preparation**: Anu Kallio, Auli Suominen, Mimmi Tolvanen, Satu Lahti; **Writing – review and editing**: Auli Suominen, Mimmi Tolvanen, Kari Rantavuori, Heidi Jussila, Linnea Karlsson, Hasse Karlsson, Satu Lahti; **Project administration**: Linnea Karlsson, Hasse Karlsson.

## CONFLICTS OF INTEREST

The authors declare no conflicts of interest.
